# Supplementary figures and images for: AI-Enhanced Lower Extremity X-Ray Segmentation: A Promising Tool for Sarcopenia Diagnosis
Source: Healthcare (Basel). 2025 Sep 30;13(19):2488. doi: 10.3390/healthcare13192488 (PMC12524675; doi:10.3390/healthcare13192488)

Figure S1. Result of semantic segmentation model.

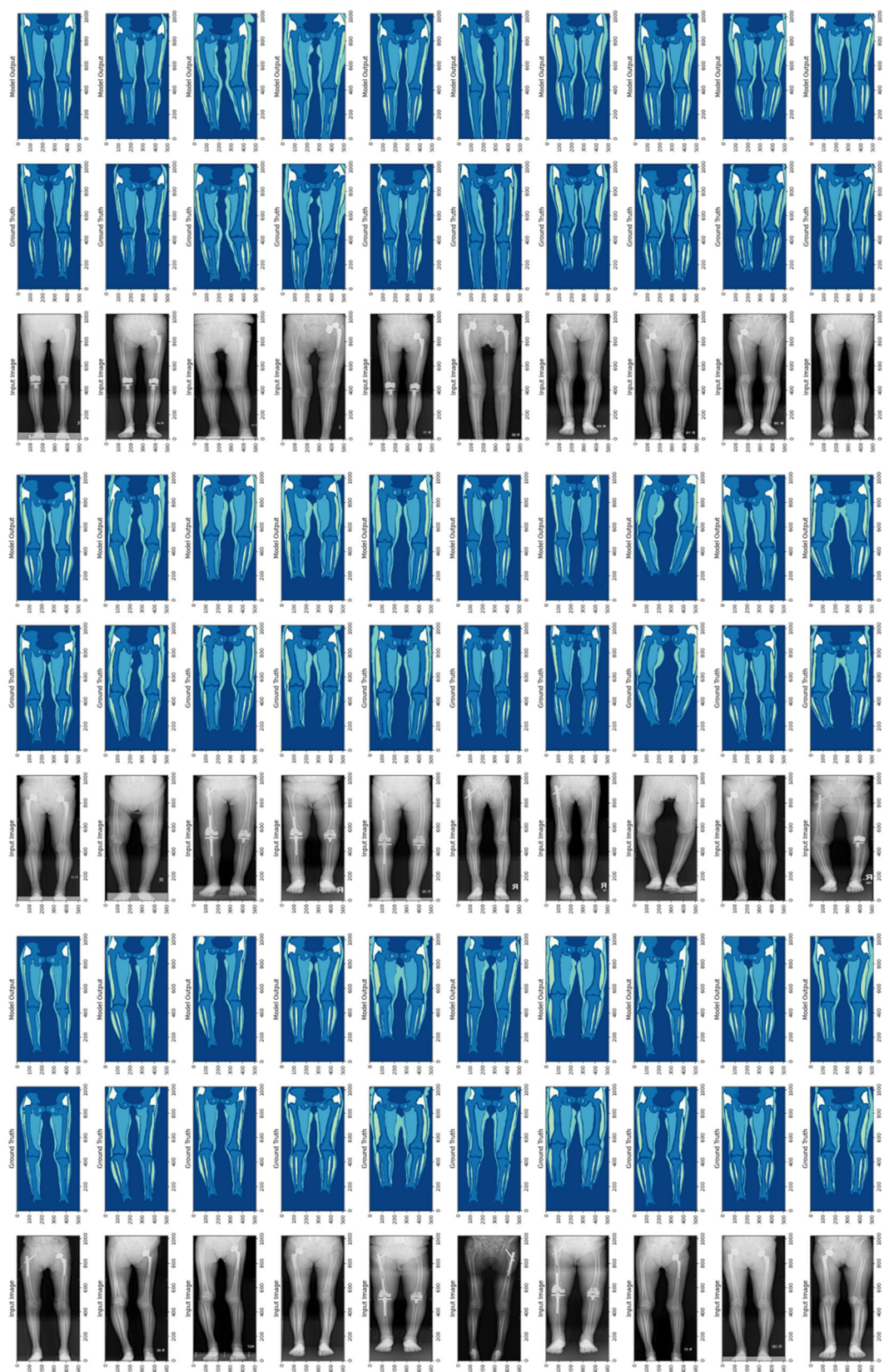

Supplement: Supplementary file 1 [file healthcare-13-02488-s001.zip › healthcare-3831840-supplementary.pdf]
